# Rethinking mobility: Life space outperforms physical activity as a correlate of health outcomes among older adults in Singapore

**DOI:** 10.1016/j.pmedr.2026.103656

**Published:** 2026-10-10

**Authors:** Sapphire Lin, Xiaohui Xin, Yee Sien Ng

**Affiliations:** a Centre for Population Health Research and Implementation, Singapore Health Services, Singapore; b Duke-NUS Medical School, Singapore; c The Institute for Digital Medicine, Yong Loo Lin School of Medicine, National University of Singapore, Singapore; d The N.1 Institute for Health, National University of Singapore, Singapore; e Health Services Research Unit, Singapore General Hospital, Singapore Health Services, Singapore; f Department of Rehabilitation Medicine, Singapore General Hospital, Singapore Health Services, Singapore

**Keywords:** Active aging, Self-rated health, Physical function, Activity space, Structural equation modeling

## Abstract

**Objective:**

Physical activity and life space are modifiable behavioral factors relevant to preventive medicine. While both are associated with health outcomes in older adults, their distinct contributions remain unclear. This study examined their associations with self-rated health and objectively measured physical performance, and tested whether effects on self-rated health were mediated by physical performance.

**Methods:**

We recruited 1118 community-dwelling older adults aged ≥50 years in Singapore between April 2022 and August 2023. Participants completed the University of Alabama at Birmingham's Life Space Assessment, the International Physical Activity Questionnaire-Short Form, and the EuroQol-Visual Analogue Scale. Physical performance was assessed using the Short Physical Performance Battery and three additional performance-based tests. Structural regression modeled direct effects on self-rated health and indirect effects via physical performance.

**Results:**

Life space emerged as a stronger predictor of both outcomes. It had both direct and indirect effects on self-rated health, whereas physical activity had only an indirect effect via physical performance.

**Conclusions:**

These findings suggest that life space may serve as a complementary or alternative indicator to traditional physical activity metrics for preventive medicine, supporting early identification and monitoring of functional decline in older adults.

## Highlights

• Life space shows stronger links to performance and self-rated health than activity.
• Life space affects health via direct and indirect paths; activity acts indirectly.
• Life space supports preventive medicine by capturing mobility and participation.

## 1 Introduction

With populations aging rapidly around the world, maintaining the health of older adults has become a priority. Preventative care is essential for mitigating the burden of age-related chronic diseases and disability (Wen and Wu, 2012). This study investigates how two modifiable behavioral factors—physical activity and life space—can support healthy aging by contributing to physical performance and self-rated health. By incorporating subjective and objective measures of health, we aim to provide a comprehensive representation of older adult health (Jung and Lee, 2024).

Although related, physical activity and life space are conceptually distinct. Physical activity involves any movement of the body driven by skeletal muscles that uses energy (World Health Organization, 2024). Life space refers to the geographical area through which a person moves in daily life over a specified period, conventionally measured with a series of concentric zones extending from the room in which one sleeps to destinations beyond the town where one lives (Bayat et al., 2021; May et al., 1985). Life space is therefore measures more than ambulation; it is an indicator of community participation as well as physical mobility.

Both physical activity and life space are established predictors of health in later life. In this study, we probe their associations with self-rated health and physical performance, and test which factor is more strongly associated with these outcomes through direct and indirect pathways. We also consider whether life space has potential as an ecologically valid biomarker of healthspan—one that complements traditional physical activity measures and supports efforts to monitor health in real-world settings (Bayat et al., 2021).

Physical activity in older adults is associated with skeletal muscle strength, muscle power, and cardiorespiratory fitness (Ramsey et al., 2021; Izquierdo et al., 2021). Physically active older adults have reduced risk of all-cause and cardiovascular mortality, recurrent falls, and disability in activities of daily living (Cunningham et al., 2020). Beyond physical benefits, physical activity is also linked to improved subjective health outcomes such as life satisfaction and well-being (World Health Organization, 2022). Accordingly, we posit:

H1: Physical activity is positively associated with (a) physical performance and (b) self-rated health of older adults.

The measurement of physical activity in older populations can be challenging (Kelly et al., 2016), and prone to both underestimation and overestimation. In fact, self-report instruments often yield floor effects, as older adults may not perceive themselves as engaging in moderate- or vigorous-intensity physical activity, and may struggle to recall or estimate engagement in physical activity (Heesch et al., 2010; Lin, 2021).

Physical activity can be assessed across three domains: (a) leisure-time, (b) occupation, education, and household, and (c) transportation (World Health Organization, 2020). However, a meta-review of the barriers and facilitators of physical activity highlighted a bias toward leisure-time activity, with less attention paid to other domains (Garcia et al., 2022). Movement beyond the home entails ambulation (e.g., walking to a bus stop, negotiating a transit interchange, moving through a shopping mall), and this incidental, transport-related activity is precisely what older adults tend not to recall when asked to report their physical activity. In light of the limitations associated with self-reports of physical activity—particularly in older adults—life space serves as a promising complementary marker of healthspan.

Larger life spaces are associated with health maintenance, functional preservation, and social engagement, whereas constricted life spaces predict mortality, morbidity, and healthcare utilization (Baker et al., 2003; Johnson et al., 2020). Beyond physical mobility, the concept also encapsulates interaction with the built and social environment, serving as a holistic ecological indicator of autonomy and functional status (Bayat et al., 2021). Indeed, out-of-home trips are associated with measures such as step counts and moderate or vigorous physical activity, and represent a plausible way to increase older adults' overall physical activity (Liu et al., 2021). Beyond objectively measured health outcomes, the evidence shows that life space mobility is associated with multiple aspects of subjectively measured health outcomes, including health-related quality of life and wellbeing (Bentley et al., 2013; Chitalu et al., 2022). Conversely, reductions in life space mobility have been linked to deterioration in quality of life and risk of depression (Rantanen et al., 2021; Tran et al., 2025). Therefore, we posit:

H2: Life space is positively associated with (a) physical performance and (b) self-rated health of older adults.

Self-rated health is shaped by a broad spectrum of factors, including objectively measured health outcomes. Prior research has shown a bidirectional relationship between physical health and subjective wellbeing, with poor physical health reducing wellbeing and vice versa (Steptoe et al., 2015). Self-reported health is associated with the number of chronic conditions (Mavaddat et al., 2014), independence in activities of daily living (Jung and Lee, 2024), and mobility (Mavaddat et al., 2011). Despite the well-established associations between objectively measured health and self-rated health, a subset of older adults display high self-rated health even in the presence of poor objective health indicators (Reinwarth et al., 2023), reinforcing that physical health is only one of the pathways to successful aging.

Building on this evidence, we model physical performance as a mediator because it is the proximal physiological consequence of movement. We acknowledge that this relationship is likely bidirectional (Dunlap et al., 2022) and that our study's cross-sectional design precludes directional inference. The model therefore tests an association and a theoretically motivated path. Specifically, we propose the following:

H3a: Physical performance is positively associated with self-rated health.

H3b: Physical performance mediates the relationship between (i) physical activity/ (ii) life space and self-rated health.

Finally, physical activity and life space are often studied separately, and there are limited studies directly comparing their relative and joint contributions for different health outcomes. In this study, we test a dual-pathway model to understand how they affect distinct health domains by posing the question: Is physical activity or life space a better predictor of older adults' physical performance and self-rated health?

## 2 Methods

### 2.1 Study design and population

We collected data from community-dwelling older adults in Singapore (*N* = 1118) between April 2022 and August 2023. Participants completed a questionnaire and a battery of physical performance tests during a single on-site session lasting approximately 1.5 h and were reimbursed 20 Singapore dollars in grocery vouchers.

We recruited participants through both traditional and digital media, targeting the general public and members of active aging centers. Eligibility was limited to individuals aged 50 years and above. We employed quota sampling to align the sample distribution with the national population in terms of age group, housing type, and frailty status (Department of Statistics, 2022). Stratification by housing type helped to ensure socioeconomic diversity.

### 2.2 Measures

Life space was measured with the University of Alabama at Birmingham's Life Space Assessment (UAB-LSA) (Baker et al., 2003), a widely used and validated instrument (Johnson et al., 2020) capturing the extent, frequency, and independence of a person's movement across different spatial zones. Participants reported whether, in the past four weeks, they had been to other rooms of their home besides the room where they sleep (level 1); an area outside their home that is still adjacent to it (level 2); places in their immediate neighborhood, other than their own yard or apartment building (level 3); places outside their immediate neighborhood but within their town (level 4); and places outside their town (level 5). Each level was weighted 1 to 5, with a “Yes” response scored accordingly and a “No” scored 0. For each level, participants also indicated the frequency of visits, scored 0 (never), 1 (less than once a week), 2 (1–3 times/week), 3 (4–6 times/week), or 4 (daily); and the level of independence, scored 1 (needed personal assistance), 1.5 (used equipment only), or 2 (no assistance or equipment). Each level score was the product of the level weight, frequency, and independence. The total life space score is the sum of the five level scores. The maximum attainable score is 8 at level one (1 × 4 × 2), 16 at level two (2 × 4 × 2), 24 at level three, 32 at level four, and 40 at level five; these sum to a total range of 0–120 (Baker et al., 2003).

Physical activity was assessed using the International Physical Activity Questionnaire - Short Form (IPAQ-SF) (IPAQ, 2005), a widely used self-report questionnaire that captures physical activity across three intensity levels: vigorous, moderate, and walking. Participants were asked to report the number of days in the past week they had engaged in each type of activity, as well as the average duration per day. Responses were used to estimate weekly physical activity by weighting time spent in each activity intensity with the estimated metabolic equivalent of task (MET). For each activity type, MET-minutes per week were calculated by multiplying the number of days, the number of minutes per day, and the corresponding MET weight: 8.0 for vigorous activity, 4.0 for moderate activity, and 3.3 for walking. These were then summed across the three activity types.

Self-rated health was assessed using the EuroQol Visual Analogue Scale (EQ-VAS) (EuroQol Research Foundation, 2019), which asks respondents to rate their overall health on the day of assessment. The scale ranges from 0 (worst imaginable health state) to 100 (best imaginable health state). This response was treated as a continuous measure.

Physical performance was assessed using the Short Physical Performance Battery (SPPB), a composite measure of lower-extremity performance comprising gait speed, balance in three positions, and the five-times sit-to-stand test (5XSST). Each component is scored 0–4 and the total ranges 0–12, with higher scores indicating better performance. Gait speed was timed over a 4-m course walked at usual pace. Balance was assessed by holding three progressively challenging standing positions—feet together, semi-tandem, and full tandem—for 10 s each. In the 5XSST, participants rose from a seated position to full standing five times as quickly as possible.

Three additional physical performance tests were administered and analyzed individually. First, the 30-Second Chair Stand Test (30CST) assessed lower limb strength and endurance by counting full chair stands completed within 30 s. Second, the Timed Up and Go (TUG) test evaluated dynamic balance, agility, and reaction time. Participants rose from a chair, walked 3 m, turned around a cone, and returned to sitting, with the faster of two trials used. Lastly, the Two-Minute Step Test (TMST) assessed cardiopulmonary endurance. Participants marched in place for two minutes; only right knee lifts reaching a wall mark at the midpoint between the iliac crest and patella were counted.

### 2.3 Statistical analysis

Structural regression was used to model both the direct effects of life space and physical activity on self-rated health, as well as their indirect effects mediated by physical performance (see Fig. 1).

**Fig. 1:**
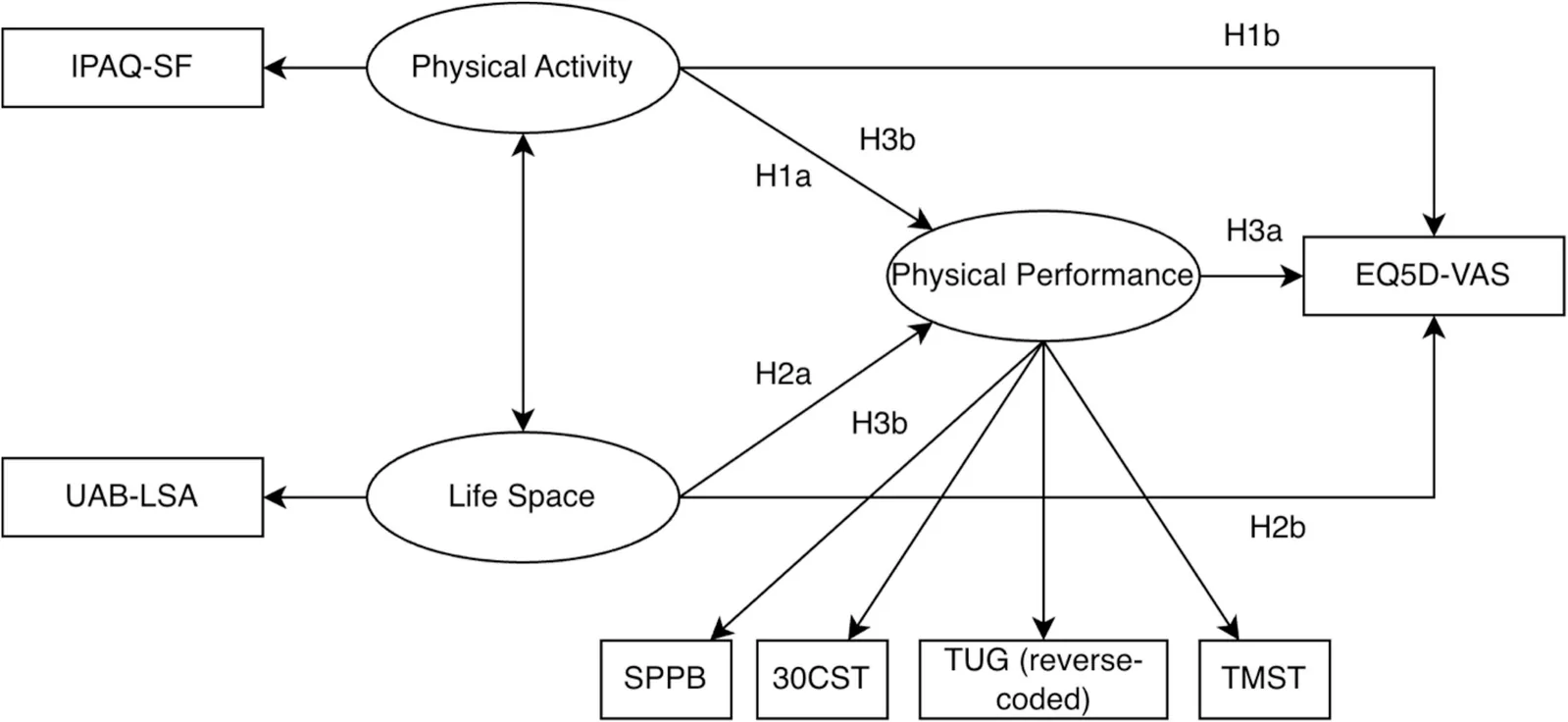
Hypothesized structural model of the associations between physical activity, life space, physical performance, and self-rated health in community-dwelling adults aged 50 years and above, Singapore, April 2022 to August 2023. *Note.* TUG: Timed Up and Go; TMST: Two-Minute Step Test; 30CST: 30-Second Chair Stand Test; SPPB: Short Physical Performance Battery; IPAQ-SF: International Physical Activity Questionnaire-Short Form; UAB-LSA: University of Alabama at Birmingham Life Space Assessment; EQ-VAS: EuroQol Visual Analogue Scale. TUG scores were reverse-coded to maintain consistent directionality.

Self-rated health was treated as an observed variable, measured by EQ-VAS. Life space and physical activity were modeled as latent constructs, measured by the corresponding observed indicators: UAB-LSA and IPAQ-SF. Physical performance was modeled as a latent construct indicated by four performance-based assessments: SPPB, 30CST, TUG, and TMST.

For the two single-indicator latent constructs (life space and physical activity), the estimated error variance of each construct was calculated by the observed variance of its indicator multiplied by the difference of one minus the reliability of the indicator (Kline, 2023). As the reliability was not directly assessed in this study, reliability values were sourced from the literature. The test-retest reliability of UAB-LSA was 0.96 (Baker et al., 2003), and that of IPAQ-SF was 0.76 based on pooled data from 12 countries (Craig et al., 2003). Life space and physical activity constructs were scaled to their respective observed scores (UAB-LSA and IPAQ-SF), while physical performance was scaled to the reverse-coded TUG scores.

Covariance coverage for all variables ranged from 0.93 to 1.00, so no imputation was performed. A robust maximum likelihood estimator was used to account for multivariate non-normality and to use all available data for model estimation. All analyses were conducted in Mplus 8.10 (Muthen and Muthen, 2017).

The SingHealth Centralised Institutional Review Board reviewed and approved this study (Reference Number 2021/2566). All participants provided written informed consent before data collection. The study met institutional guidelines for the protection of human subjects with respect to safety and privacy, and the research team adhered to the Personal Data Protection Act and the Human Biomedical Research Act.

## 3 Results

Participants (*N* = 1118) were between 50 and 88 years old, and the mean age was 63.77 (*SD* = 7.64). More than half (67.98%) were female. Table 1 presents the descriptive statistics and correlation matrix of the observed variables.

**Table 1:** Descriptive statistics and correlations among measures of physical performance, physical activity, life space, and self-rated health in community-dwelling adults aged 50 years and above, Singapore, April 2022 to August 2023.

Observed Variables	TUG_Rev	TMST	30CST	SPPB	IPAQ-SF	UAB-LSA	EQ-VAS
**Correlations**							
TUG_Rev	1						
TMST	0.56	1					
30CST	0.55	0.66	1				
SPPB	0.57	0.60	0.59	1			
IPAQ-SF	0.10	0.11	0.11	0.10	1		
UAB-LSA	0.26	0.19	0.19	0.20	0.19	1	
EQ-VAS	0.22	0.21	0.17	0.18	0.09	0.16	1

**Descriptive Statistics**							
Mean	34.21	91.05	16.32	11.42	2.37	90.21	83.89
*SD*	2.46	20.27	5.21	1.39	2.15	18.13	12.10
Skewness	−5.04	−0.49	0.94	−3.35	2.03	−0.46	−1.28
Kurtosis	47.26	0.69	1.27	14.84	6.16	0.46	3.80
*N*	1115	1044	1040	1118	1088	1118	1118

*Note.* TUG_Rev: Timed Up and Go (reverse-coded); TMST: Two-Minute Step Test; 30CST: 30-Second Chair Stand Test; SPPB: Short Physical Performance Battery; IPAQ-SF: International Physical Activity Questionnaire-Short Form; UAB-LSA: University of Alabama at Birmingham Life Space Assessment; EQ-VAS: EuroQol Visual Analogue Scale. TUG scores were reverse-coded to maintain consistent directionality; IPAQ-SF metabolic equivalent of task were divided by 1000 to keep the variance of this variable below the 1,000,000 limit of the Mplus software.

The structural regression model tested the relationships between life space and physical activity (as predictors), self-rated health (as the outcome), and physical performance (as a mediator). The model explains 7.60% of the total variance in self-rated health.

The model as specified in Fig. 1 is a good fit to the data as evidenced by the fit statistics: ꭓ^2^(11) = 26.43, *p* = .01; Comparative Fit Index (CFI) = 0.99; Tucker-Lewis Index (TLI) = 0.98; Root Mean Square Error of Approximation (RMSEA) = 0.04; and Standardized Root Mean Square Residual (SRMR) = 0.02. These values suggest that the hypothesized model adequately represents the observed data, with CFI and TLI above the recommended threshold of 0.90, and RMSEA and SRMR below 0.06 and 0.08 respectively (Bentler, 1992; Hu and Bentler, 1999).

In Fig. 2, we summarize the findings for all tested hypotheses by indicating the β value for all paths. The results showed that physical activity was significantly associated with physical performance (β = 0.10, *p* < .01), supporting H1a, but its direct association with self-rated health was not significant (β = 0.05, *p* = .19), thus H1b was not supported. Life space was positively associated with both physical performance (β = 0.25, *p* < .01) and self-rated health (β = 0.10, *p* = .01), supporting H2a and H2b. Physical performance was positively associated with self-rated health (β = 0.22, *p* < .01), supporting H3a.

**Fig. 2:**
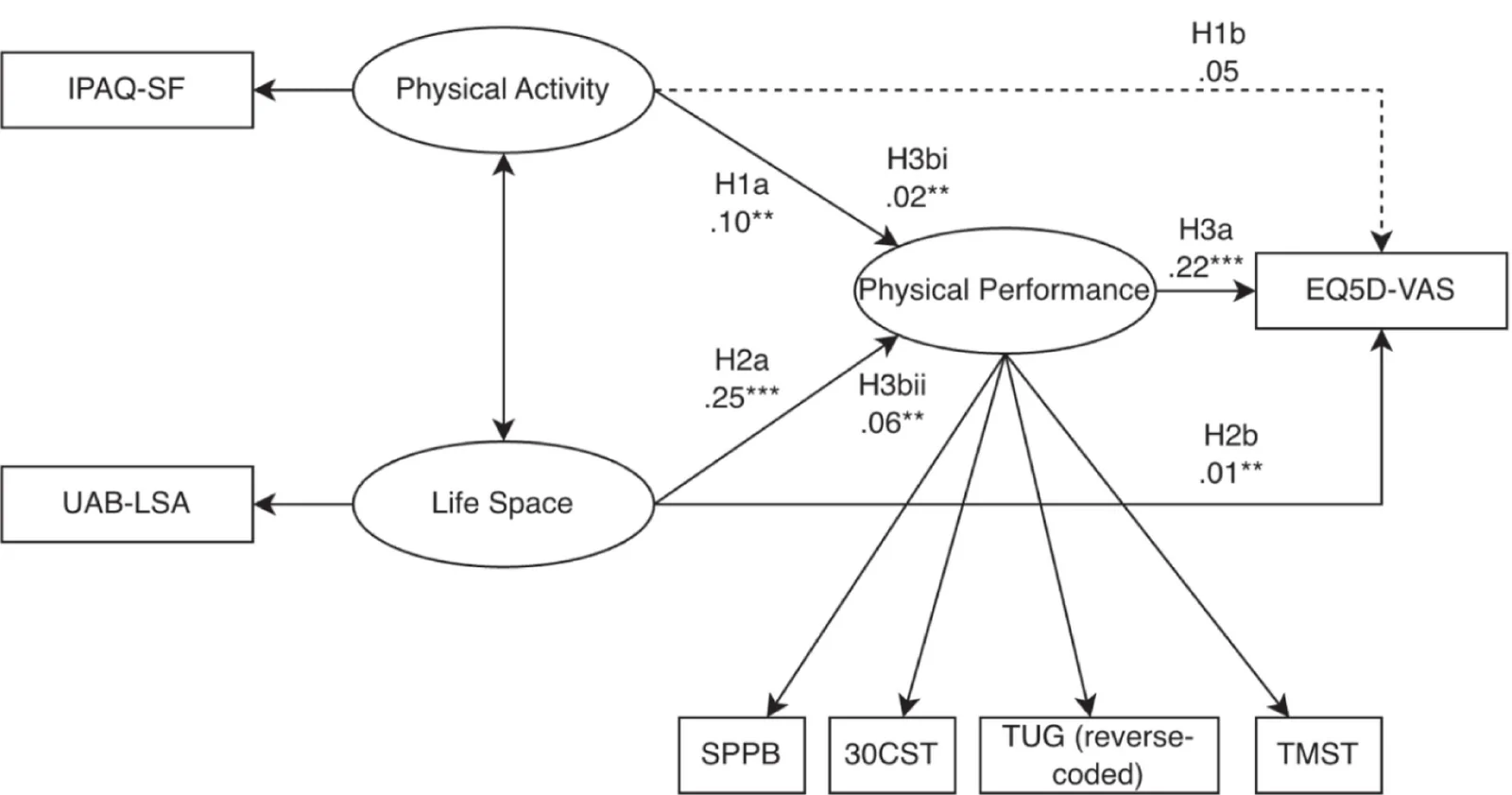
Structural model with standardized parameter estimates for community-dwelling adults aged 50 years and above, Singapore, April 2022 to August 2023. *Note.* TUG: Timed Up and Go; TMST: Two-Minute Step Test; 30CST: 30-Second Chair Stand Test; SPPB: Short Physical Performance Battery; IPAQ-SF: International Physical Activity Questionnaire-Short Form; UAB-LSA: University of Alabama at Birmingham Life Space Assessment; EQ-VAS: EuroQol Visual Analogue Scale. TUG scores were reverse-coded to maintain consistent directionality.

Mediation analyses indicated that life space has both direct and indirect effects on self-rated health whereas physical activity has only an indirect effect on self-rated health, through enhancing physical performance (see Table 2). An increase in one *SD* of life space score is associated with an increase of 0.15 *SD* of self-rated health. Two-thirds of this effect comes directly from life space, and the other one-third is mediated by physical performance. In contrast, the effect of physical activity on self-rated health is much smaller, with an increase of one *SD* of physical activity score being associated with an increase of only 0.02 *SD* of self-rated health mediated by physical performance. Comparatively, life space emerged as a stronger predictor of physical performance and self-rated health than physical activity.

**Table 2:** Direct, indirect, and total associations of life space and physical activity with self-rated health in community-dwelling adults aged 50 years and above, Singapore, April 2022 to August 2023.

Parameter	*b*	*SE*	β
**Direct effect**			
Life space	0.07**	0.02	0.10
Physical activity	0.32	0.24	0.05

**Indirect effect through physical performance**			
Life space	0.04**	0.01	0.06
Physical activity	0.14**	0.05	0.02

**Total effect (direct + indirect)**			
Life space	0.10***	0.03	0.15
Physical activity	0.46	0.25	0.07

**Disturbance variances**			
EQ-VAS	135.15***	10.29	0.92
Physical performance	3.04**	0.95	0.92

*Note.* EQ-VAS: EuroQol Visual Analogue Scale. *p < .05, **p < .01, ***p < .001. Estimates are unstandardized (*b*) and standardized (β) coefficients from a structural regression model fitted with maximum likelihood estimation with robust standard errors.

## 4 Discussion

This study compared physical activity and life space as correlates of physical performance and self-rated health among older adults. Both factors affected self-rated health indirectly via their effects on physical performance. However, life space showed stronger associations with self-rated health, demonstrating significant direct and indirect associations. In contrast, physical activity did not show a significant direct association with self-rated health, though it contributed indirectly through physical performance. Though the associations we observed were modest in magnitude, these results suggest that life space may serve as a useful complement to physical activity as a behavioral indicator of healthspan in older populations.

We recommend that self-report instruments measuring physical activity in older adults be administered with greater care and clarification. Since travel-related activity falls under the broader category of physical activity, it appears paradoxical that physical activity had weaker associations with health. The distribution of reported activity among our participants was not unusual (IPAQ, 2005), with most classified as moderately or highly active. Despite this, life space was found to be more strongly associated with both health outcomes, suggesting gaps in how older adults understand or report their physical activity.

Indeed, instruments like the IPAQ-SF may be prone to measurement error in older populations due to recall challenges, poor estimation of duration or intensity, and misinterpretation of what constitutes physical activity (Heesch et al., 2010). For example, many older adults do not consider themselves to be engaging in vigorous-intensity activities—defined in the IPAQ-SF as those that cause breathlessness—while such activities are given high MET weights of 8.0 in the IPAQ-SF. In addition, the wide array of instruments measuring physical activity has resulted in great variations in activity estimates (Silveira et al., 2022). These limitations highlight the need for more age-sensitive physical activity assessments. Our findings are consistent with life space offering a more intuitive measure of activity-related contributors to healthspan in this age group. Researchers and clinicians may wish to consider life space assessments alongside traditional physical activity measures for older adults.

Beyond identifying methodological improvements, this study contributes to a broader understanding of the distinct yet complementary roles of activity and mobility in older adult health. These findings suggest value in interventions that also address life space expansion, rather than relying solely on conventional physical activity promotion. Given that some older adults may avoid leisure-time physical activity due to pain, discomfort, or concerns about exacerbating existing conditions (Lin, 2021), interventions that enhance life-space mobility offer a more feasible and empathetic pathway to improving health outcomes in later life.

This has meaningful implications for public health, policy, and urban design. It underscores the importance of promoting social and community participation as a key determinant of healthy aging. Designing environments and programs that enable older adults to move more freely and safely through their neighborhoods is a plausible complement to encouraging structured exercise and warrants evaluation. In addition, the association between physical performance and self-rated health suggests that physicians could provide older adults with focused prescriptions on lower limb training.

This study has several limitations. First, both IPAQ-SF and UAB-LSA are self-report instruments subject to recall bias and measurement error when compared to objective tools such as accelerometry or global positioning system tracking. Nevertheless, we selected these instruments to enable a parallel comparison between physical activity and life space, both measured in self-report format. Importantly, IPAQ-SF and UAB-LSA are widely used and validated tools for assessing their respective constructs (Baker et al., 2003; Johnson et al., 2020; IPAQ, 2005).

Second, the structural model accounted for only 7.60% of the variance in self-rated health. The associations we noted were modest too. These findings reflect the inherently complex nature of self-rated health, which is shaped by a wide range of factors—for example, mental well-being, cognitive status, income adequacy, and social relationships—that were not included in analysis. The purpose of this study was not to develop a comprehensive predictive model but to compare the contributions of physical activity and life space.

Third, our sample spans a broad age range, and adults aged 50 may differ substantially from those aged 75 in employment, morbidity, and mobility—differences that a single model may not fully capture. Age may therefore be a common cause of both life-space mobility and self-rated health. Nevertheless, the quota-sampling strategy helped ensure that the sample broadly reflected Singapore's population in terms of age, gender, and socioeconomic status.

## 5 Conclusions

We suggest that life space merits consideration in both research and practice as a contextually relevant indicator of older adults' health. Unlike metrics that focus on physical exertion alone, life space also reflects social engagement, environmental accessibility, and real-world functional capacity. In this sample, life space was associated with both objective physical performance and self-rated health, and more consistently so than physical activity, though the associations were small. By reflecting how individuals interact with and navigate their environments, life space offers a measure that may help to guide interventions, inform policy, and support healthy, independent living in later life. Longitudinal work is needed to establish whether expanding life space improves health, or whether better health simply enables a larger life space.

## CRediT authorship contribution statement

**Sapphire Lin:** Writing – review & editing, Writing – original draft, Validation, Project administration, Methodology, Investigation, Data curation, Conceptualization. **Xiaohui Xin:** Writing – original draft, Formal analysis, Data curation. **Yee Sien Ng:** Writing – review & editing, Supervision, Methodology, Funding acquisition, Conceptualization.

## Ethics declaration

Written informed consent to take part in the study and to publish the article has been obtained from all participants or their legal representatives. The privacy rights of participants have been observed.

This study was performed in compliance with relevant laws, regulatory frameworks and guidelines where the research took place. This study was approved by the SingHealth Centralised Institutional Review Board (Approval No. CIRB Reference Number: 2021/2566).

## Funding

This research/project is supported by the 10.13039/501100001381National Research Foundation, Singapore, and Ministry of National Development, Singapore, under its Cities of Tomorrow R&D Programme (CoT Award COT-H1-2020-1). Any opinions, findings and conclusions or recommendations expressed in this material are those of the author(s) and do not reflect the views of National Research Foundation, Singapore, and Ministry of National Development, Singapore.

## Declaration of competing interest

The authors declare that they have no known competing financial interests or personal relationships that could have appeared to influence the work reported in this paper.

## Data Availability

The data generated during and/or analyzed during the current study are not currently publicly available.
